# miRNA Expression Profiles in Isolated Ventricular Cardiomyocytes: Insights into Doxorubicin-Induced Cardiotoxicity

**DOI:** 10.3390/ijms25105272

**Published:** 2024-05-12

**Authors:** Yohana Domínguez Romero, Gladis Montoya Ortiz, Susana Novoa Herrán, Jhon Osorio Mendez, Luis A. Gomez Grosso

**Affiliations:** 1 Doctorate in Biotechnology Program, Faculty of Sciences, Universidad Nacional de Colombia, Bogotá 111321, Colombia; lydominguezr@unal.edu.co; 2 Molecular Physiology Group, Sub-Direction of Scientific and Technological Research, Direction of Public, Health Research, National Institute of Health, Bogotá 111321, Colombia; gmontoya@ins.gov.co (G.M.O.); snovoa@ins.gov.co (S.N.H.); josoriome@unal.edu.co (J.O.M.); 3 Master in Biochemistry Program, Department of Physiological Sciences, Faculty of Medicine, Universidad Nacional de Colombia, Bogotá 111321, Colombia; 4 Department of Physiological Sciences, Faculty of Medicine, Universidad Nacional de Colombia, Bogotá 111321, Colombia

**Keywords:** doxorubicin, cardiotoxicity, cardioprotection, miRNAs, KATP, SIRT1, FOXO1, GSK3B

## Abstract

Doxorubicin (DOX), widely used as a chemotherapeutic agent for various cancers, is limited in its clinical utility by its cardiotoxic effects. Despite its widespread use, the precise mechanisms underlying DOX-induced cardiotoxicity at the cellular and molecular levels remain unclear, hindering the development of preventive and early detection strategies. To characterize the cytotoxic effects of DOX on isolated ventricular cardiomyocytes, focusing on the expression of specific microRNAs (miRNAs) and their molecular targets associated with endogenous cardioprotective mechanisms such as the ATP-sensitive potassium channel (KATP), Sirtuin 1 (SIRT1), FOXO1, and GSK3β. We isolated Guinea pig ventricular cardiomyocytes by retrograde perfusion and enzymatic dissociation. We assessed cell morphology, Reactive Oxygen Species (ROS) levels, intracellular calcium, and mitochondrial membrane potential using light microscopy and specific probes. We determined the miRNA expression profile using small RNAseq and validated it using stem-loop qRT-PCR. We quantified mRNA levels of some predicted and validated molecular targets using qRT-PCR and analyzed protein expression using Western blot. Exposure to 10 µM DOX resulted in cardiomyocyte shortening, increased ROS and intracellular calcium levels, mitochondrial membrane potential depolarization, and changes in specific miRNA expression. Additionally, we observed the differential expression of KATP subunits (ABCC9, KCNJ8, and KCNJ11), FOXO1, SIRT1, and GSK3β molecules associated with endogenous cardioprotective mechanisms. Supported by miRNA gene regulatory networks and functional enrichment analysis, these findings suggest that DOX-induced cardiotoxicity disrupts biological processes associated with cardioprotective mechanisms. Further research must clarify their specific molecular changes in DOX-induced cardiac dysfunction and investigate their diagnostic biomarkers and therapeutic potential.

## 1. Introduction

One of the most widely used and effective treatments for cancer is Doxorubicin (DOX); however, previous studies have shown that this therapy can induce cytotoxicity [1,2]. Doxorubicin-induced cardiotoxicity (DIC) defines a direct or indirect adverse effect of this chemotherapy that can lead to cardiac dysfunction and even heart failure during both treatment and weeks, months, or years after treatment [3,4,5,6]. Furthermore, there is a significant increase in the risk of death from heart failure observed in patients treated with anthracyclines such as DOX [7,8].

Despite its widespread clinical use, there is limited knowledge about the direct effects of DOX on cardiac cells at the cellular and molecular level. However, researchers have proposed several mechanisms that may be involved in DOX-induced heart failure, such as the generation of Reactive Oxygen Species (ROS), increased lipid peroxidation, deregulation of intracellular calcium concentration, alteration of cell membrane integrity, and inhibition of topoisomerase II (Top2), which may lead to apoptosis [6,9]. Furthermore, researchers have observed alteration of mitochondrial function as a possible mechanism by which DOX induces cardiomyocyte death [10,11]. The guinea pig is a popular model used in scientific research to study isolated cardiomyocytes [12,13]. This animal model exhibits remarkable similarities with humans regarding molecular and cellular physiology and electrophysiology [14,15,16]. Doxorubicinol, a metabolite derived from the transformation of DOX within the cardiomyocyte, has been shown to affect excitation–contraction coupling in isolated guinea pig ventricular cardiomyocytes. These responses are comparable to those observed in humans, underscoring their fundamental importance in the study of anthracycline-induced cardiotoxicity [17].

Research in experimental models has shown that exposure to DOX causes morphological and structural changes in the heart, including dilation of the atria, elongation and hypertrophy of ventricles, vacuolization, loss of myofibrils, dilation of the sarcoplasmic reticulum, and alterations in mitochondria [11,18,19,20,21]. Despite discontinuing treatment, researchers have observed a progressive decrease in ventricular ejection fraction, indicating that cardiac injury may persist even after discontinuation of DOX and involve cardioprotection mechanisms [22].

In this context, it is crucial to understand the mechanisms of DOX cardiotoxicity, identify cardioprotective agents, and develop non-invasive biomarkers to detect the risk of DIC as miRNAs. Research on the differential expression of miRNAs is emerging as a promising field to understand the mechanisms associated with DIC and to identify biological markers that can prevent chemotherapy-induced cardiac toxicity [23]. Our study explores the direct effects of doxorubicin (DOX) exposure on microRNA (miRNA) expression and some putative molecular targets to elucidate the cellular and molecular alterations in cardiomyocytes that may predispose someone to heart failure. Our focus lies specifically on investigating changes in miRNA expression associated with doxorubicin-induced isolated cardiomyocyte injury, with particular attention to changes in pivotal miRNAs involved in regulating critical molecules involved in endogenous cardioprotective mechanisms, such as KATP, FOXO1, and SIRT1, among others. This research aimed to contribute to understanding DIC mechanisms and provide relevant information for developing prediction, prevention, and treatment strategies for DOX-induced cardiac toxicity.

## 2. Results

### 2.1. Doxorubicin Causes Damage to Guinea Pig Ventricular Cardiomyocytes

**Figure 1:**
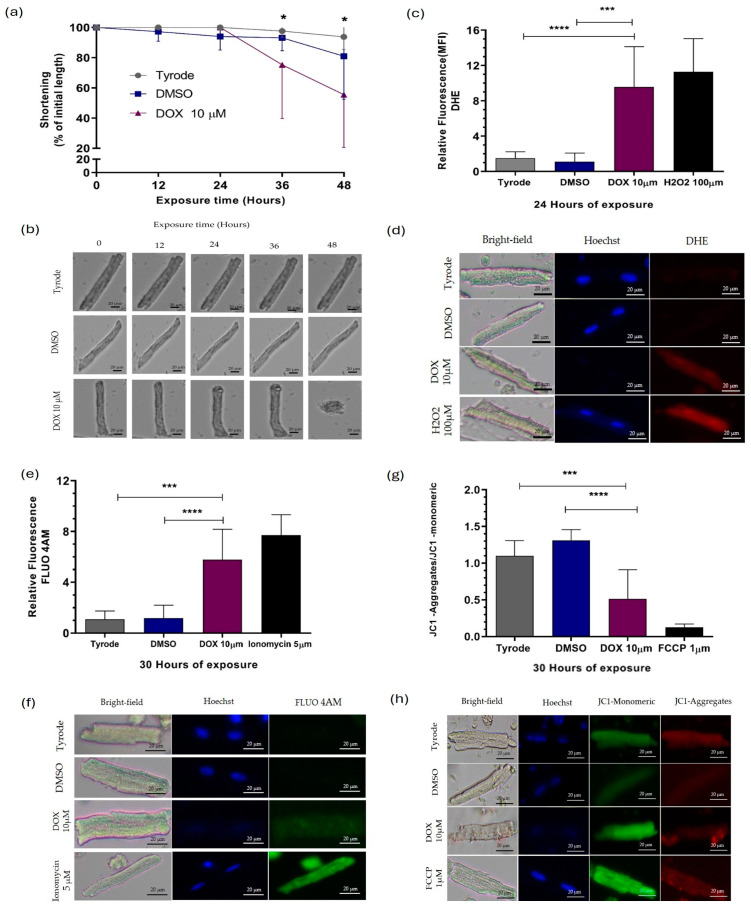
DOX-induced injury in ventricular cardiomyocytes of *Cavia porcellus*. We exposed isolated cardiomyocytes to 10 μM DOX: (**a**) % shortening and (**b**) representative photographs of the shortening of cardiomyocytes during 48 h of exposure to DOX, (**c**) ROS levels analyzed with DHE, (**d**) representative photographs of ROS production in cardiomyocytes exposed to DOX for 22 h, (**e**) intracellular calcium levels analyzed with FLU4AM and (**f**) representative photographs of calcium levels in cardiomyocytes exposed to DOX for 30 h. (**g**) Mitochondrial membrane potential (ΔΨm) analyzed with JC1 and (**h**) representative photographs of ΔΨm in cardiomyocytes exposed to DOX for 30 h. Nuclear counterstain with DAPI (blue), quantification of expression by mean fluorescence intensity (MFI), Data were analyzed using two-way ANOVA parametric statistical test (*p* < 0.05, *n* = 10 cardiomyocytes per group)—representative graphs of three different experiments. Significance level of statistical findings: (*) *p* < 0.0332, (***) *p* < 0.0002, significant (****) *p* < 0.0001.

To assess DOX-induced cardiomyocyte injury, we evaluated DOX concentration and treatment duration by measuring percent shortening. Various DOX concentrations (1–15 μM) were applied to cardiomyocytes for 48 h, and the percentage of shortening was evaluated (Figure S1a). Healthy cardiomyocytes exhibit an elongated shape (80–120 μm long, 20–30 μm wide), whereas injured cardiomyocytes shorten and hypercontract, exhibiting a rounded morphology, as shown in Figure 1a. This morphological assessment served as the first viability indicator after DOX exposure [24]. Cells exposed to 1 and 5 μM DOX showed no significant reduction in length compared to controls. However, exposure to 10 μM, 12 μM, and 15 μM DOX for 36, 35, and 21 h resulted in significant shortening (75.3%, 75.1%, and 77.5% reduction, respectively). These results established the optimal conditions (10 μM, 1–30 h) for evaluating DOX-induced cardiomyocyte injury (Figure S1a–d).

In our cardiomyocyte model, we selected the concentration of cellular treatment based on the intention to imitate the average dose of DOX used in patients, which is known for causing cardiotoxicity. Supplementary Figure S1 depicts the effect of concentrations ranging from 1 to 15 µM DOX on the shortening percentage, directly proportional to cell viability. We selected 10 µM DOX as the optimal concentration, determined by the fact that it did not significantly decrease cell viability over the average life of our model (48 h). This concentration was chosen to characterize the lesion in terms of ROS production, intracytoplasmic Ca^2+^ levels, and mitochondrial membrane potential at different time points (1, 12, 24, and 30 h), as shown in Supplementary Figures S2–S4, respectively.

We assessed the impact of direct exposure to DOX on cardiomyocytes; we exposed cells to 10 µM of DOX for varying periods. As shown in Figure 1, cardiomyocytes experienced a decrease in length after approximately 36 h of exposure, resulting in a 75.3% shortening (Figure 1a,b). This shortening was accompanied by a significantly increased ROS level 12, 24, and 30 h after treatment, with increased percentages of 75.3%, 75.1%, and 77.5%, respectively (Figure 1c,d and Figure S2). Interestingly, at 30 h of exposure to DOX, intracellular calcium (Ca^2+^) levels also increased (Figure 1e,f and Figure S3), with a 3.8-times increase, and the mitochondrial membrane was significantly depolarized (Figure 1g,h and Figure S4), with percentages of decrease in the JC1 aggregates/JC1 monomeric of 61.5%. These findings indicate that DOX influences individual cardiomyocytes’ structure, viability, and functioning of the mitochondria, which aligns with results from other studies conducted on various cell models [10,11,20].

To analyze the effect of DOX exposure on the expression of miRNAs and their potential molecular targets, we selected two critical time points—1 h and 22 h. We chose these specific time points based on our initial observations of DOX-induced cellular injury (Figure 1a–h). During the first hour of exposure, we did not observe any structural signs of cellular injury, oxidative stress, elevated intracytoplasmic Ca^2+^ levels, or mitochondrial damage. However, around the 22 h mark, we noticed a significant increase in ROS production. Despite this increase in oxidative stress, we did not observe any compromise in cardiomyocyte viability or responsiveness. By focusing on these specific exposure times, it was possible to differentially study the early and late responses to DOX exposure in our experimental model.

The absence of nuclear staining observed in cardiomyocytes exposed to DOX in Figure 1d,f,h was attributed to the intricate mechanism of intercalation, wherein DOX molecules insert themselves between the base pairs of double-stranded DNA [9]. This process may effectively obstruct Hoechst dye’s binding sites on the DNA molecule. Hoechst dye typically binds to adenine/thymine-rich DNA regions in the minor groove, facilitating nuclear staining [25]. However, because DOX can compete for these binding sites on the DNA molecule, Hoechst dye cannot bind to the DNA and stain the nucleus. Therefore, the lack of Hoechst staining in the nuclei of DOX-exposed cardiomyocytes underscores the profound impact of DOX on DNA structure and integrity, highlighting a pivotal aspect of its mechanism of cardiotoxicity.

### 2.2. Small RNAseq and Differential Expression Analysis of miRNAs

**Figure 2:**
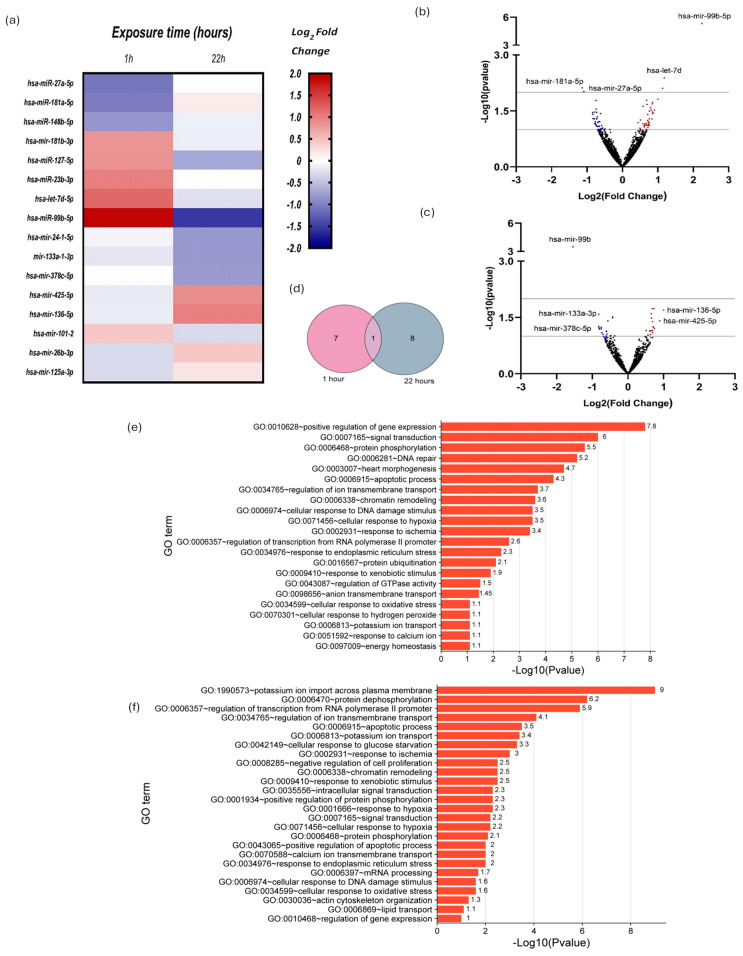
The expression miRNAs from *Cavia porcellus* isolated ventricular cardiomyocytes exposed to 10 µM DOX for 1 and 22 h. (**a**) Heat map of 16 expressed miRNAs obtained by small RNA-seq versus vehicle control cells (DMSO); red, expression up; blue, expression down; and white, indicator of no variation. Differential expression by using the DESeq2 program. (**b**,**c**) Volcano plots of miRNAs from cardiomyocytes exposed to 10 µM DOX for 1 and 22 h, respectively. Blue dots represent downregulated miRNAs, and red dots represent upregulated miRNAs. We only included miRNAs with –log10 (*p* value) ≥ 1 and log2 fold change ≥ 0.8 and ≤ −0.8 in blue or red dots. (**d**) Venn diagrams showing the numbers of miRNAs differentially expressed at 1 h and 22 h. (**e**,**f**) The related biological process with DOX-induced cardiotoxicity –log10 (*p* value) ≥ 1.

Before visible damage appears in the cardiomyocyte due to exposure to DOX, the cell can detect the damage and start a series of molecular changes. These responses include changes in the expression of different genes, including those that regulate the expression of other genes like miRNAs. For this reason, we used small RNA sequencing to determine the expression levels of miRNAs in cardiomyocytes exposed to 10 µM DOX for 1 and 22 h and to detect differences in expression compared to unexposed cardiomyocytes. From the normalized data and using DESeq2, we found that some miRNAs changed their expression in DOX-treated cardiomyocytes compared to untreated ones at 1 and 22 h of exposure to DOX. We generated a heat map from the small RNA sequencing data, illustrating the miRNA expression patterns in ventricular cardiomyocytes after exposure to 10 µM DOX (DOX) for 1 and 22 h compared to unexposed cells (Figure 2a and Table 1 and Table 2, respectively). The heatmap displayed about sixteen miRNAs expressed relative to vehicle control (DMSO) cells, with red indicating upregulation, blue indicating downregulation, and white indicating no change (Figure 2a). We conducted differential expression analysis using the DESeq2 program.

Volcano plots illustrated miRNA expression changes post 1 h and 22 h DOX exposure (Figure 2b,c). Blue dots depict downregulation, and red depict upregulation. Included were miRNAs meeting –log10 (*p* value) ≥ 1, with log2 fold changes ≥ 0.8 or ≤ −0.8, spotlighting pivotal miRNAs for deeper analysis of DOX-induced cardiac molecular changes. Notably, miR-99b-5p and miR-27a-5p exhibited significant expression differences post 1 h exposure (Figure 2b,c): miR-99b-5p with a 1.91-fold increase and miR-27a-5p with a −1.09-fold decrease. After 22 h, miR-99b-5p decreased (−1.46-fold), contrasting with miR-27a-5p increase (Figure 2a,b). To enrich notable miRNA identification, those with significant up or downregulation (≥0.8 or ≤−0.8-fold changes) were included (Figure 2c and Table 1 and Table 2).

The Venn diagram illustrates the count of miRNAs exhibiting differential expression, encompassing 1 h (depicted by the pink circle) and 22 h of exposure to DOX (represented by the gray circle). Within the intersection of these two sets lies the singular miRNA showing differential expression in both conditions, identified as miR-99b-5p (Figure 2d).

We conducted Gene Ontology (G.O.) term enrichment analysis for biological processes using the target genes of the identified miRNAs with changes in their expression related to DOX-induced cardiotoxicity at 1 h and 22 h of exposure. The G.O. enrichment analysis was performed with a –log10 (*p* value) ≥ 1 value. Figure 2e–f summarizes the enrichment analysis of G.O. terms of interest.

These findings indicated that DOX directly influences the reciprocal regulation of specific miRNAs in isolated guinea pig ventricular cardiomyocytes. This influence may activate several critical biological processes, including regulating gene expression, DNA repair mechanisms, cellular responses to DNA damage, and oxidative stress. The enrichment analysis of G.O. terms for biological processes supports this suggestion, indicating the involvement of these processes at both 1 and 22 h of exposure to DOX (Figure 2e–f, respectively).

### 2.3. Validation of miRNA Differential Expression by RT-qPCR Stem-Loop

**Figure 3:**
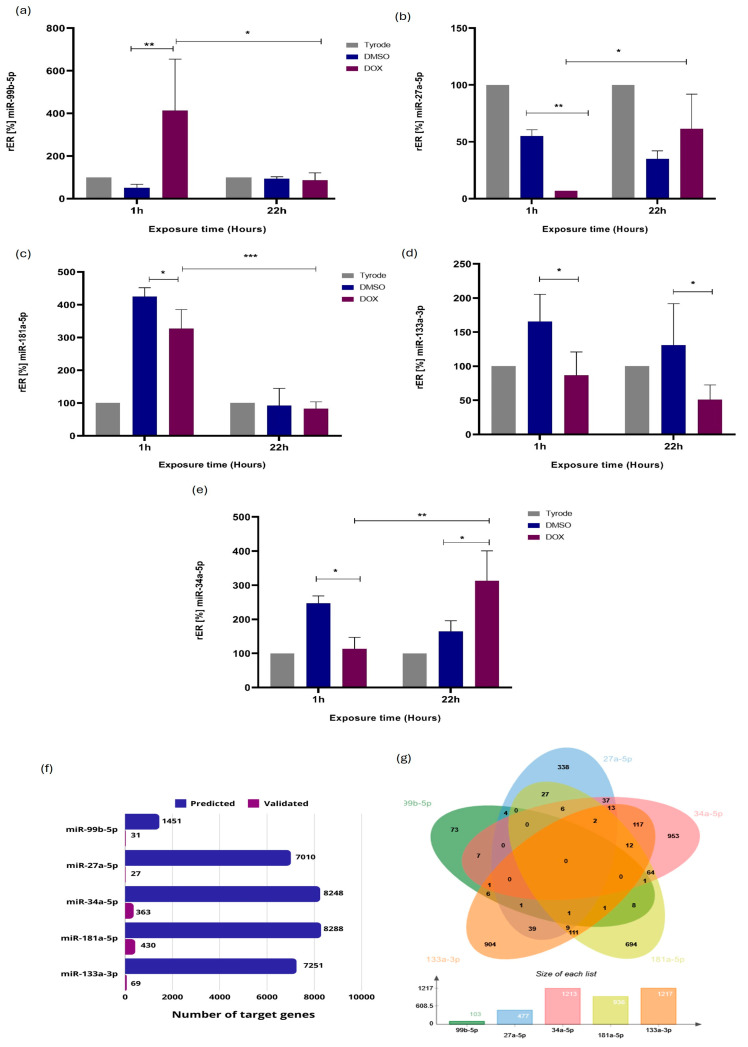
Validation of the differential expression of some miRNAs associated with *Cavia porcellus* ventricular cardiomyocytes’ response to DOX-induced injury. Relative expression ratios (rERs) of miRNAs by RT-qPCR stem-loop for (**a**) miR-99b-5p, (**b**) miR-27a-5p, (**c**) miR-181a-5p, (**d**) miR-133a-3p, and (**e**) miR-34a-5p (*n* = 3). (**f**) Several target genes predicted and validated to miRNAs are evaluated. (**g**) Venn diagrams showing the numbers of target genes predicted and validated shared between the miRNAs analyzed. Data were analyzed using a two-way ANOVA parametric statistical test (*p* < 0.05, *n* = 3)—representative graphs of three experiments. Significance level of statistical findings: (*) *p* < 0.0332, (**) *p* < 0.0021 (***) *p* < 0.0002. We performed target gene analysis in CSmiRTar. (http://cosbi4.ee.ncku.edu.tw/CSmiRTar/search) (accessed on 27 March 2024). At least two databases support the predicted target genes, and the average normalized score (ANS) is ≥0.2.

We validated miRNA expression via RT-qPCR Stem-Loop assays following RNAseq analysis. Selected miRNAs included miR-27a-5p, miR-99b-5p, miR-133a, miR-181a-5p, and miR-34a-5p (Figure 3). miR-34a-5p was included based on our previous studies. Relative differential expression (rER) confirmed differential patterns. At 1 h post-exposure (hpe) to 10 µM DOX, cardiomyocytes significantly downregulated miR-27a-5p (5-fold), miR-133a-3p (2-fold), miR-181a-5p (1.3-fold), and miR-34a-5p (2.5-fold), while miR-99b-5p increased (8-fold). At 22hpe, miR-34a-5p increased (1.7-fold), and miR-133a-3p decreased (3-fold) compared to controls (Figure 3). These findings aligned with NGS for miR-99b-5p, miR-27a-5p, miR181a-5p (1 hpe), and miR-133a-3p (22 hpe). However, miR-99b-5p and miR-133a-3p showed discrepancies between the RT-qPCR and NGS results. Notably, miR-99b-5p exhibited significant differential expression at 1 hpe and 22 hpe, corroborating RNAseq data (Figure 2d).

We investigated whether there were differences in miRNA expression levels between 1 hpe and 22 hpe. We found that miR-99b-5p, miR-27a-5p, and miR-181a-5p at 22 hpe were differentially expressed concerning cardiomyocytes at 1 hpe (Figure 3). The results agree with the data obtained by RNAseq regarding miR-99b-5p and miR-27a-5p; however, this agreement does not extend to miR-181a-5p (Figure 2a and Figure 3c). The results described in Figure 3f,g provide valuable insights into the miRNA–target interactions evaluated in the study. In Figure 3f, the number of target genes predicted and validated for the analyzed miRNAs indicates the impact of the regulatory roles of these miRNAs. A higher count suggests a more impacted biological process. Figure 3g, additionally, the Venn diagrams depicting the overlap of predicted and validated target genes among the analyzed miRNAs reveal shared targets between multiple miRNAs. This overlap signifies potential cooperative or synergistic effects regulating these common target genes, highlighting the complexity and interconnectedness of miRNA-mediated regulatory networks and molecular pathways.

To understand DOX-induced molecular mechanisms, we predicted miRNA targets using G.O. enrichment analysis (Figure 2d,f). At 1 and 22 h post-exposure, DOX affected various biological processes (–log10 (*p* value) ≥ 1), including DNA damage response pathways, activating repair mechanisms against drug-induced genotoxicity. Upregulated apoptotic pathways indicated increased programmed cell death in cardiac tissue, contributing to observed cardiac dysfunction and ROS-induced oxidative stress, disrupting cellular balance and causing tissue damage. Additionally, inflammatory pathway activation exacerbated tissue damage and impaired cardiac function post-DOX exposure. These findings highlight the multifaceted nature of DOX-induced cardiotoxicity involving DNA damage, apoptosis, oxidative stress, and inflammation, potentially leading to cardiac dysfunction.

### 2.4. ATP-Sensitive Potassium Channel (KATP) Coding Genes Are Expressed Differently in Cardiomyocytes Injured by Direct Exposure to DOX

**Figure 4:**
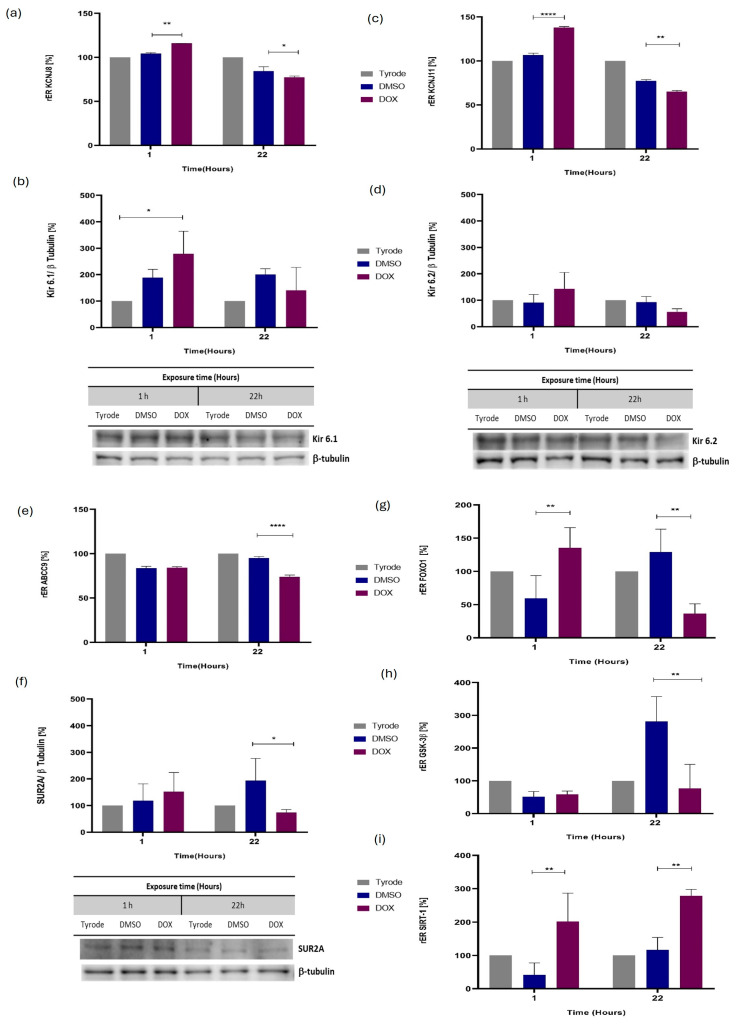
The expression of genes and their protein products is associated with *Cavia porcellus* ventricular cardiomyocytes’ response to DOX-induced injury. Relative expression radius (rER) of target genes (**a**) KCNJ8, (**c**) KCNJ11, (**e**) ABCC9, (**g**) FOXO1, (**h**) GSK3β, and (**i**) SIRT-1 by RT-qPCR and protein products (**b**) Kir 6.1, (**d**) Kir 6.2, and (**f**) SUR2A by Western blot. Data were analyzed using a two-way ANOVA parametric statistical test (*p* < 0.05, *n* = 3)—representative graphs of three experiments. Significance level of statistical findings: (*) *p* < 0.0332, (**) *p* < 0.0021, significant (****) *p* < 0.0001.

Considering the results obtained regarding DOX-induced injury in cardiomyocytes, the changes in miRNA expression, and the fact that miRNAs act by acting on mRNA molecules, we investigated possible molecular targets related to this injury. Previous studies showed a significant role of KATP channels in cardioprotection, as cellular stress activates them [26,27]. Thus, we evaluated the gene expression levels of several genes in cardiomyocytes exposed to 10 μM DOX for 1 h and 22 h by RT-qPCR. KATP channels comprise three functional subunits: SUR2A, Kir6.1, and Kir6.2, which the ABCC9, KCNJ8, and KCNJ11 genes encode, respectively [28]. To determine whether DOX alters the expression of KATP-encoding genes, we evaluated mRNA levels in cardiomyocytes exposed or not to 10 μM of DOX at the previously defined time points. As shown in Figure 4, cardiomyocytes treated with DOX for 22 h exhibited decreased mRNA expression of the KCNJ8 (2.1-fold decrease) (Figure 4a), KCNJ11 (1.7-fold decrease) (Figure 4c) and ABCC9 (2.6-fold decrease) genes (Figure 4e) compared to the control cardiomyocytes. At the protein level, cardiomyocytes exposed to DOX did not exhibit significant changes in Kir6.1 levels compared to the vehicle (Figure 4b). At the protein level, among the three subunits composing KATP, SUR2A exhibited decreased expression after 22 h of exposure to DOX (Figure 4f), consistent with the observations made at the mRNA level (Figure 4e). We observed no statistically significant variation in protein levels for Kir6.1 (Figure 4b) and Kir6.2 (Figure 4d) despite the differential expression of mRNA for the genes encoding them (Figure 4a,c, respectively).

### 2.5. Differential mRNA Expression for Additional miRNA Molecular Targets, Including GSK3β, SIRT1, and FOXO1

We comprehensively searched for other potential molecular targets of our differentially expressed miRNAs associated with DOX cardiotoxicity or cardioprotection. Our search identified three interesting molecules for evaluation: Forkhead Box O1 (FOXO1), glycogen synthase kinase-3 beta (GSK3β), and Sirtuin 1 (SIRT1). Researchers have reported that FoxO1 participates in the regulation of oxidative stress in rat cardiomyocytes [29]. FoxO1 regulates glucose and fatty acid metabolism in cardiac mitochondrial dysfunction in type 1 diabetes [30]. Additionally, FoxO1 increases the expression of the Kir 6.1, Kir 6.2, and SUR2A subunits of the KATP channel [31]. Our RT-qPCR results showed a significant increase in FOXO1 expression 1hpe to DOX (2.4-fold increase), which was followed by a statistically significant decrease at 22 h post-exposure (4-fold decrease) (Figure 4g), suggesting a significant role in DOX-mediated cardiotoxicity. According to the scientific literature, miR-99b-3p, but not miR-99b-5p, targets glycogen synthase kinase-3 beta GSK-3β [32], which was associated with the inhibition of electron transport chain subunits, leading to ROS production and ATP depletion. Although we did not detect miR-99b-3p in our study, we evaluated GSK3b expression [33]. Interestingly, at 22 h post-exposure to DOX, GSK3β mRNA exhibited a statistically significant decrease (3.5-fold decrease) (Figure 4h). Finally, researchers have proposed that SIRT1 influences cardiac function through its association with regulating energy metabolism and cardiac hypertrophy and its demonstrated protective effect against oxidative stress [34]. Notably, our results showed a significant increase in Sirt1 mRNA levels following direct exposure to DOX at both 1 (5-fold increase) and 22 h (2.8-fold increase) compared to the control (Figure 4i); we witnessed similar findings for FOXO1, indicating cellular response to DOX-induced stress. For a proper analysis of the expression of these molecules, their confirmation at the protein level is necessary by Western blot.

### 2.6. miRNA Functional Analysis and Phenotype-Associated Molecules Network

To elucidate the biological impact of differentially expressed miRNAs that explain the cellular changes associated with DOX-induced injury in cardiomyocytes, we developed a functional and network analysis of the selected miRNAs, their reported target genes, and phenotype-relevant proteins such as KATP channel subunits and cardioprotective-related proteins. We used the ClueGO/CluePedia plugin from Cytoscape (ClueGO v2.5.10, CluePedia v. 1.5.10, Cytoscape v. 3.9.1) [35,36,37]. We analyzed the up and downregulated miRNAs as clusters (up in red and down in blue), obtained the validated reported target genes, and performed a pathway enrichment analysis, selecting those associated with the observed phenotype. For 1 hpe, we obtained 1496 target genes from 10 miRNA and 735 target genes for 22 hpe from 7 miRNA retrieved from mirTarBase and miRecords databases (enrichment of all MTI for each miRNA, up to 300 genes, score ≥ 0.6) (see Tables S1 and S2 CluePedia). To the list of miRNA–target interactions (MTIs), we included proteins of interest such as KATP subunits KCNJ11, KCNJ8, and ABCC9 and cardioprotection-related ones such as SIRT1 and GSK3B, its transcription factor FOXO1, and its experimental change in expression.

Pathway enrichment analysis was performed using Reactome, Wikipathway, and the KEGG database. Specific for the 1 hpe up cluster (target gene up, miRNA down) are pathways such as “Formation of Senescence-Associated Heterochromatin Foci (SAHF)” (corrected *p*-value = 7 × 10^−5^, 62% associated genes), “Apoptosis-induced DNA fragmentation” (corrected *p*-value = 2 × 10^−3^, 62% associated genes), and “Oxidative stress-induced senescence” (corrected *p*-value = 3 × 10^−7^, 34 genes, 27% associated genes), and for the down cluster (target gene down, miRNA up) are “Somatroph axis (GH) and its relationship to dietary restriction and aging” (corrected *p*-value = 3 × 10^−4^, 100% associated genes) and “Sphingolipid metabolism in senescence” (corrected *p*-value = 4 × 10^−2^, 10 genes, 36% associated genes). For the 22 hpe results, the only one specific for the up cluster is the “AGE/RAGE pathway” (corrected *p*-value = 1 × 10^−2^, 12 genes, 18% associated genes). In contrast, for the down cluster, the highlights are “ATP-sensitive potassium channels” Reactome pathway (corrected *p*-value =2 × 10^−3^, 4 genes, 100% associated genes), “POU5F1 (OCT4), SOX2, NANOG repress genes related to differentiation” (corrected *p*-value = 4 × 10^−2^, 3 genes, 100% associated genes), “DNA Damage/Telomere Stress Induced Senescence” (corrected *p*-value = 3 × 10^−5^, 17 genes, 21% associated genes), in addition to the above pathways (“Oxidative Stress Induced Senescence”, corrected *p*-value = 2 × 10^−6^, 23 genes, 18% associated genes), and “Somatroph Axis (GH)” (corrected *p*-value = 6 × 10^−4^, 83% associated genes) as a non-specific pathway (see Tables S1 and S2 ClueGO).

**Figure 5:**
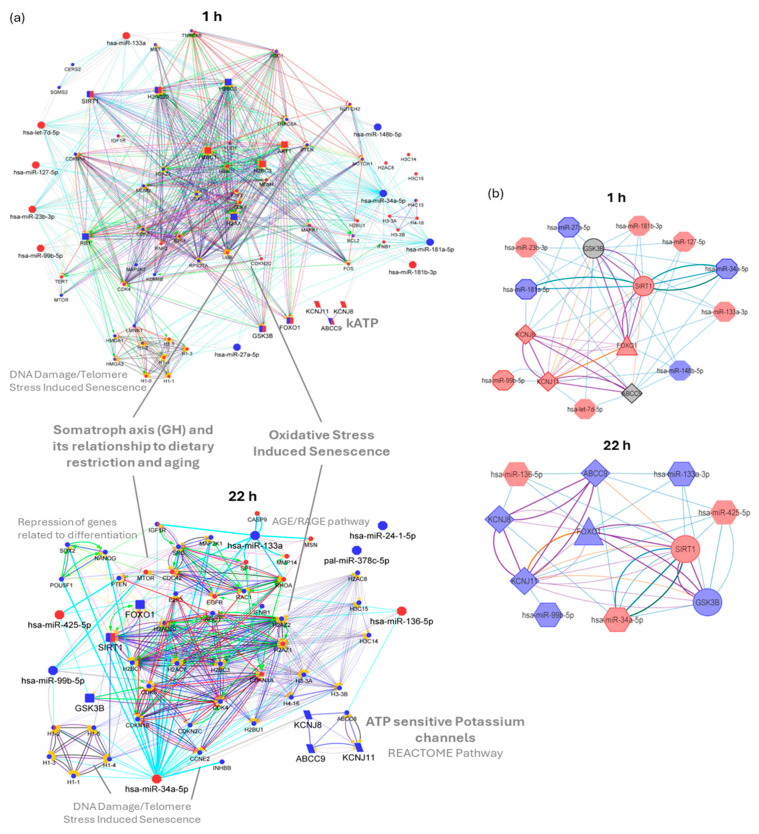
miRNA–target interactions (MTIs) and protein–protein interaction (PPI) network for DOX-altered miRNAs and genes. (**a**) CluePedia visualization of miRNA, proteins of interest, and relevant validated target genes obtained from miRecord and mirTarBase. (**b**) Summary network of experimentally evaluated miR and genes, where thick border node represents confirmation by RT-PCR. Blue and red nodes correspond to downregulated and upregulated molecules, respectively. The label font size and node size highlight the selected molecules. Target genes from phenotype-relevant pathways in circles, miRNAs in octagons, cardioprotective-related proteins in rounded rectangles (**a**) or circles (**b**), network-relevant proteins (degree ≥ 4) in rectangles, KATP channel subunits in parallelograms (**a**) or diamonds (**b**). Edge width is proportional to Kappa score and color edges depict interaction type: (**a**) predicted MTI in grey, validated MTI in aquamarine (miRTarBase) and blue (miRecords), activation in green, binding in blue, catalysis in purple, expression in yellow, inhibition in red, reaction in black, PTM in fuchsia. For (**b**), color edges depict interaction source: CSmiRTar in blue, validated miRTarbase and miRecord in aquamarine and green, respectively, PPI STRING in purple, and literature in orange. Networks were built with Cytoscape 3.9.1.

These pathways were selected, and their genes were used as input, together with altered miRNA and proteins of interest as those regulated by more than four miRNA (degree ≥ 4) (see Tables S1 and S2 MTI PPI). Figure 5a shows the obtained molecular networks combining protein–protein interaction (PPI) and miRNA–target interaction (MTI). For 1 hpe, network analysis reveals high degree nodes such as transcription factor RB1 (belonging to Senescence-Associated Heterochromatin Foci (SAHF) and Sphingolipid metabolism in senescence) and with the lowest average shortest path length and highest betweenness centrality such as hsa-miR-34a-5p, RB1, hsa-let-7d-5p, AKT1, SIRT1, has-miR-133a, hsa-miR-23b-3p, H2AC20, H2BC1/3/5, and TNRC6B (belonging to Oxidative Stress-Induced Senescence). For 22 hpe, among the nodes with higher degrees are H2AZ1/2, SIRT1, the transcription factor CDK4, hsa-miR-34a-5p, and H2BC1/3, which are also the nodes with the lowest average shortest path length and highest betweenness centrality, together with KCNJ8 and hsa-miR-133a. These miR and genes found by network-based analysis control the information flow in the networks and are critical elements in the altered pathway. Finally, we built an MTI and PPI summary network consolidating the experimental results for expressing miRNAs and target genes in cardiomyocytes exposed to DOX, including predicted and validated MTIs and STRING PPIs (Figure 5b).

## 3. Discussion

The effectiveness of DOX in treating cancer underscores its potential to induce cardiotoxicity, resulting in cardiac dysfunction and heart failure [8,38]. Despite its widespread use, there remains a need for a deeper understanding of how DOX directly impacts cardiac cells at both the cellular and molecular levels.

Proposed mechanisms for DOX-induced cardiotoxicity include generating ROS, disrupting intracellular calcium homeostasis, mitochondrial dysfunction, and induction of apoptosis [9,39,40,41]. Experimental studies have demonstrated structural alterations in the heart following DOX exposure, indicating cardiac injury post-treatment [38]. Given the imperative need to comprehend DOX cardiotoxicity and identify cardioprotective strategies, research on miRNAs has gained prominence [42]. This study investigated how direct exposure to DOX affects miRNA expression and its targets, mainly focusing on miRNAs involved in cardioprotective mechanisms such as KATP, SIRT1, and FOXO1 on the guinea pig experimental model, which offers a valuable platform for investigating changes in the expression of miRNAs and some of their targets in response to doxorubicin-induced cardiomyocyte injury [43], providing insights into human cardiac physiology and potential therapeutic strategies. By describing some molecular changes induced by DOX in cardiomyocytes, this research contributes to understanding the mechanisms underlying DOX-induced cardiotoxicity and identifying potential targets for prevention, diagnosis, and intervention strategies.

The results demonstrate that DOX exposure induces damage to guinea pig isolated ventricular cardiomyocytes. Initial assessments based on cell morphology revealed that cardiomyocytes exposed to concentrations of 10 μM or higher for durations ranging from 21 to 36 h exhibited significant shortening, indicating loss of viability. Further investigation showed that exposure to 10 μM DOX for 36 h resulted in a 75.3% reduction in cell length, accompanied by increased levels of ROS and intracellular calcium. Additionally, mitochondrial membrane depolarization suggests disruption of cellular bioenergetics and metabolism. These findings highlight the detrimental effects of DOX on cardiomyocyte structure and function, consistent with previous research conducted on various cell models [9,44]. Overall, the study underscores the importance of understanding the mechanisms underlying DIC to develop strategies for mitigating its adverse effects on cardiac health.

Our results indicate a direct effect of DOX on guinea pig isolated ventricular cardiomyocytes, as evidenced by cell shortening, increased levels of ROS, intracellular calcium overload, and mitochondrial membrane depolarization. These findings are consistent with loss of viability and proposed mechanisms of cardiac cytotoxicity observed in different cellular models [39,41,45]. The alteration of calcium homeostasis, with an intracellular increase, is associated with the induction of shortening and subsequent contracture of the cardiomyocyte. Changes in cytoplasmic Ca^2+^ establish a link between plasma membrane depolarization and contraction, referred to as excitation–contraction coupling [46].

The increase in ROS production observed in our experimental model may be related to the oxidation–reduction processes that DOX undergoes upon entering the cardiomyocyte through passive diffusion. These reactions generate ROS as by-products, promoting an oxidative environment that could be lethal for the cardiomyocyte, especially given its high mitochondrial density and energy consumption [47]. DOX can bind to cardiolipin on the mitochondrial membrane, resulting in a change in mitochondrial membrane potential. This process uncouples the electron transport chain, which, in turn, promotes the production of ROS [47,48,49,50,51]. Furthermore, increased ROS production is associated with elevated cytoplasmic Ca^2+^ levels, achieved through the stimulation of Ca^2+^ release from the sarcoplasmic reticulum via activation of the ryanodine receptor and disruption of Ca^2+^ scavenging mechanisms in cardiomyocytes [45,52,53].

Additionally, as previous studies have shown, DOX can directly elevate cytoplasmic Ca^2+^ levels by interacting with molecules involved in Ca^2+^ release, such as the SERCA2 protein [53]. This direct interaction of DOX with Ca^2+^ regulatory mechanisms in cardiac cells underscores its potential contribution to cardiomyocyte injury. Consequently, this disruption leads to dysregulation of intracellular Ca^2+^ homeostasis, ultimately promoting cardiomyocyte apoptosis [54,55,56]. The findings of this study support the previous studies about the interaction between oxidative stress and calcium dysregulation in the context of doxorubicin-induced cardiotoxicity.

Preserving mitochondrial membrane potential is essential for maintaining cardiomyocyte mitochondrial function, especially for the functioning of the electron transport chain and oxidative phosphorylation to generate ATP [57]. Therefore, depolarization of the mitochondrial membrane affects energy production and promotes cardiomyocyte death [58]. Our results are consistent with other works that have linked mitochondrial membrane depolarization to the opening of the mitochondrial Permeability Transition Pores (mPTPs) by molecules such as calcium and ROS [54] and the initiation of apoptosis by promoting the release of proapoptotic factors such as cytochrome c and Apoptosis-Inducing Factor (AIF) [55]. Therefore, the increase in ROS and intracellular calcium levels observed in this study after 12 and 30 h of exposure to DOX, respectively, could suggest the involvement of ROS and calcium in mPTP opening and mitochondrial depolarization in the context of DOX-induced injury.

Exposure to DOX for isolated cardiomyocytes may initiate molecular changes in cardiomyocytes preceding visible lesions, including alterations in gene expression, notably miRNAs. We used small RNA sequencing to compare miRNA expression in cardiomyocytes exposed to 10 µM DOX for 1 and 22 h with untreated cells. After being treated with DOX, there are significant changes in miRNA expression among cardiomyocytes. About sixteen miRNAs with changed expression patterns were displayed through a heat map, and DESeq2 analysis confirmed these changes. Differential expression analysis revealed significant miR-99b-5p upregulation after 1 h of DOX exposure but downregulation after 22 h. Previous studies suggest miR-99b-5p has a role in reducing cardiac hypertrophy and dysfunction [59]. Its putative targets include ARID3A, IGF1R, MFGE8, MTOR, RAVER2, and RAVER2, which are involved in various biological processes [59,60]. Conversely, miR-27a-5p overexpression after 22 h of DOX exposure is linked to cardioprotective mechanisms and inhibition of autophagy-related 7 (Atg7 [61]). These findings underscore the intricate role of miRNAs in mediating responses to DOX-induced cardiotoxicity and their potential diagnosis markers and therapeutic targets for their adverse effects.

Volcano plots illustrate miRNA expression changes post 1 and 22 h of DOX exposure. Notably, miR-99b and miR-27a show significant differential expression after 1 h, with miR-99b increasing and miR-27a decreasing. However, after 22 h, miR-99b decreases while miR-27a increases. MiRNAs with significant expression changes (log2 fold changes ≥ 0.8 or ≤−0.8) are identified for further investigation. These findings reveal dynamic alterations in miRNA expression in response to DOX-induced damage. DOX exposure likely activates signaling pathways that modulate miRNA expression levels. For example, oxidative stress triggered by DOX could stimulate the activity of transcription factors involved in regulating miRNA expression. Moreover, the observed changes in miRNA expression may signify an adaptive response by the cells to bolster their antioxidant defenses or activate pathways associated with cellular repair and survival in response to DOX-induced injury. Further exploration of the precise molecular mechanisms driving these changes is necessary to grasp their implications in DOX-induced cardiotoxicity comprehensively.

The Venn diagram shows miRNA expression at 1 and 22 h of DOX exposure, revealing miR-99b-5p as the only miRNA with altered expression in both conditions. Gene Ontology (G.O.) enrichment analysis explored the biological processes these miRNAs affect in DOX-induced cardiotoxicity. Significance was determined by –log10 (*p* value) ≥ 1. The results (Figure 2e,f) summarize enriched G.O. terms, suggesting DOX’s direct influence on the reciprocal regulation of specific miRNAs in guinea pig ventricular cardiomyocytes. This influence may activate critical biological processes, including gene expression regulation, DNA repair, and responses to oxidative stress. The enrichment analysis of G.O. terms for biological processes supports this suggestion, indicating the involvement of these processes at both 1 and 22 h of exposure to DOX.

The study validates miRNA expression by RT-qPCR stem-loop assays and confirms the results of RNAseq analysis for five miRNAs (Figure 3). Cardiomyocytes show significant miRNA expression changes at 1 and 22 h post-exposure (hpe). In particular, miR-99b-5p increases consistently at both time points, consistent with the RNAseq results. However, there are discrepancies for miR-181a-5p and miR-133a-3p. While miR-99b-5p and miR-27a-5p show consistent expression patterns between methods, inconsistencies may be due to differences in sensitivity and specificity. Variations in RNA extraction, reverse transcription, PCR amplification efficiencies, sample heterogeneity, and miRNA processing rates may affect expression measurements. In addition, differences in data normalization methods between RNAseq and RT-qPCR analyses may contribute to discrepancies in expression patterns. Despite these discrepancies, the consistent expression patterns of miR-99b-5p and miR-27a-5p across both methods provide confidence in their reliability. The agreement between miR-99b-5p expression levels and RNAseq results at both 1 hpe and 22 hpe further supports the robustness of these findings in response to DOX-induced injury. Further analysis reveals that miR-99b-5p, miR-27a-5p, and miR-181a-5p exhibit differential expression between 1 hpe and 22 hpe. These results provide insights into the temporal dynamics of miRNA expression in response to DOX-induced injury.

The changes in miRNA expression reflect the complex cellular responses to DOX-induced stress over time. Initial exposure may activate genes associated with cardioprotective mechanisms, leading to the upregulation of specific miRNAs. However, prolonged exposure may cause a shift in cellular response and a downregulation of miRNAs involved in regulatory processes. The targets and functions of these miRNAs may vary depending on the duration of DOX exposure, highlighting the need for further research to understand the mechanisms and functional implications of DOX-induced cardiotoxicity.

Additionally, analyzing predicted and validated target genes for the miRNAs sheds light on their regulatory roles in specific biological processes. The overlap of target genes among miRNAs underscores potential cooperative or synergistic effects in regulating common targets, highlighting the complexity of miRNA-mediated regulatory networks.

G.O. enrichment analysis further elucidates potential molecular mechanisms underlying DOX-induced cardiotoxicity, revealing the involvement of various biological processes such as DNA damage response, apoptosis, oxidative stress, and inflammation. These findings underscore the multifaceted nature of DOX-induced cardiotoxicity and provide valuable insights for developing molecular diagnosis markers and therapeutic strategies to mitigate its adverse effects on cardiac function.

The differential expression of mRNA for potential molecular targets associated with CID reveals insights into the underlying mechanisms of cardiac alterations. We identified KATP, FOXO1, GSK3β, and SIRT1 as potential targets of our differentially expressed miRNAs.

To assess the impact of DOX on the expression of KATP-encoding genes, we conducted an RT-qPCR analysis of mRNA levels in cardiomyocytes exposed to 10 μM DOX for 1 h and 22 h. The KATP channels consist of three functional subunits encoded by the ABCC9, KCNJ8, and KCNJ11 genes, corresponding to SUR2A, Kir6.1, and Kir6.2 [62,63]. Our findings indicate that cardiomyocytes treated with DOX for 22 h exhibit decreased mRNA expression of KCNJ8, KCNJ11, and ABCC9 genes compared to control cells. There are no significant changes in Kir6.1 levels at the protein level, whereas SUR2A displays decreased expression after 22 h of DOX exposure, consistent with mRNA results. However, the protein levels of Kir6.2 do not show significant variation despite changes in mRNA expression. These results imply that DOX exposure modulates the expression of KATP-encoding genes in cardiomyocytes, potentially influencing the function of KATP channels and contributing to DIC. Possible explanations for why we observed no statistically significant variation in protein levels for Kir6.1 (Figure 4b) and Kir6.2 (Figure 4d), despite the differential expression of mRNA for the genes encoding them (Figure 4a, c, respectively), could include post-transcriptional modifications, protein stability, translation efficiency, or the presence of regulatory factors affecting protein synthesis or degradation.

Additionally, discrepancies between mRNA and protein levels may arise due to differences in their turnover rates, cellular localization, or the involvement of complex regulatory mechanisms, such as microRNA-mediated post-transcriptional regulation or protein–protein interactions. Our study evaluated KATP expression regarding miRNA regulation as potential a gene expression regulator. Further investigation is warranted to elucidate the underlying mechanisms responsible for these observations. Understanding how doxorubicin treatment affects KATP channel activity is essential to comprehend the mechanisms of doxorubicin-induced cardiotoxicity.

FOXO1 regulates oxidative stress and metabolic pathways in cardiomyocytes [30,64,65]. Our results show a significant increase in FOXO1 expression at 1 h post-exposure to DOX, followed by a decrease at 22 h, suggesting its involvement in DOX-mediated cardiotoxicity. GSK3β plays a role in electron transport chain regulation and ROS production [32,33,66]. Interestingly, GSK3β mRNA significantly decreased at 22 h post-exposure to DOX, indicating a potential link between miR-99b-mediated regulation and DOX-induced ROS production.

SIRT1 modulates cardiac function and protects against oxidative stress [34,67,68,69]. Our findings reveal a significant increase in SIRT1 mRNA levels following DOX exposure at both 1 and 22 h, suggesting its involvement in DOX-induced alterations similar to FOXO1. Recent studies have shown that miR-34a-5p regulates SIRT1 expression in response to DOX. Our findings indicate that DOX treatment alters the expression of miR-34a-5p and SIRT1, although their expression levels do not correlate [70]. Given the lack of direct association between these two molecules, further studies are necessary to understand their potential relationship.

DOX exposure may trigger signaling pathways that regulate SIRT1 gene expression. For instance, oxidative stress induced by DOX could activate transcription factors that promote SIRT1 expression. Additionally, the observed increase in SIRT1 mRNA levels may reflect an attempt by the cells to enhance their antioxidant defense mechanisms or activate pathways involved in cellular repair and survival in response to DOX-induced damage. Further investigation into the specific molecular mechanisms underlying these changes is warranted to understand their full implications in DOX-induced cardiotoxicity.

The obtained functional and regulatory networks reveal the high connection among the studied genes, and the enriched pathways help to explain the observed DOX-induced phenotype (Figure 5). This systems biology approach is required, considering the potential cooperative or synergistic effects of altered miRNAs (Figure 3g). The proteins selected from enriched pathways are possibly associated with the DOX-induced injury phenotype and could represent key targets to evaluate in further studies. Notably, at 22 hpe, all the subunits of the KATP, analyzed as the “ATP sensitive Potassium channels” Reactome Pathway, are downregulated, determined by database mining or experimentally in the present study. The network analysis highlights genes such as hsa-miR-34a-5p, RB1, hsa-let-7d-5p, AKT1, SIRT1, hsa-miR-133a, hsa-miR-23b-3p, H2AC20, H2BC1/3/5, and TNRC6B at 1 hpe, and H2AZ1/2, SIRT1, the transcription factor CDK4, hsa-miR-34a-5p, and H2BC1/3 at 22 hpe, as crucial components in the regulatory networks. These genes control the flow of information in the networks, regulate associated pathways, and represent potential therapeutic targets. The molecular networks obtained for 22 hpe uncover the possible effect of altered miRNAs and the deleterious effects of DOX at that time. The specific enriched pathways correlate with the observed phenotype, and their associated genes and miRNAs offer novel candidates for further study and insights into understanding the molecular mechanism behind the doxorubicin-induced cardiotoxicity. The results suggest the involvement of signaling pathways in adaptation to cardiomyocyte stress conditions, while computational analyses highlight the complexity of miRNA regulation and its association with cardiotoxic progression. These insights provide novel candidates for further research and deepen our understanding of impaired cardiomyocyte function and metabolism in the context of doxorubicin-induced cardiotoxicity.

The definition of cancer therapy-related cardiac dysfunction (CTRCD) has evolved, emphasizing the need for new biomarkers like microRNAs (miRNAs) for early detection. Medical professionals now classify CTRCD based on troponin levels, global longitudinal strain (GLS) changes, and left ventricular ejection fraction (LVEF). Mild CTRCD includes troponin elevation or >15% GLS change with LVEF ≥ 50%, moderate involves a 10-point LVEF drop to 40–49%, and severe is LVEF < 40%. Recent guidelines recommend initiating beta-blockers and ACEIs for mild CTRCD to prevent progression, supported by a class IIa recommendation and level of evidence B. Incorporating miRNAs as biomarkers could improve early detection and management of CTRCD [71].

Our findings reveal the differential expression of mRNA for FOXO1, GSK3β, and SIRT1 in response to DOX exposure, emphasizing their potential roles in DOX-induced cardiotoxicity and cardiac alterations. Our study explores the direct effects of DOX exposure on miRNAs expression and some putative molecular targets to elucidate the cellular and molecular alterations in cardiomyocytes that may predispose one to heart failure. Our focus lies specifically on investigating changes in miRNA expression associated with doxorubicin-induced isolated cardiomyocyte injury, with particular attention to changes in pivotal miRNAs involved in regulating critical molecules involved in endogenous cardioprotective mechanisms, such as KATP, FOXO1, and SIRT1, among others.

Our study confirms that DOX induces cardiotoxicity by disrupting several biological processes, including oxidative stress, mitochondrial dysfunction, viability pathways, inflammation, and autophagy dysregulation. These disruptions collectively compromise the heart’s ability to withstand injury, ultimately leading to the development of cardiac dysfunction and heart failure in patients undergoing DOX-based chemotherapy. Additionally, our in silico regulatory network analysis and functional enrichment using the miRNAs identified in our study and their molecular targets suggest that DOX may interfere with endogenous cardioprotective mechanisms. This hypothesis implies that DOX’s impact extends beyond the recognized pathways, potentially impairing the heart’s resilience to injury and increasing the risk of cardiac dysfunction and heart failure in affected individuals.

However, it is essential to note that studying isolated cardiomyocytes may only partially represent the complex interactions within intact tissues or organs. While providing valuable insights into cellular responses, isolated cardiomyocytes do not capture the holistic effects of DOX on the heart or within the context of the entire cardiovascular system. Therefore, while the experimental setup allows for a detailed examination of cardiomyocyte-specific responses, further studies are warranted to elucidate the broader effects of DOX at tissue and organ levels. Integrating findings from isolated cardiomyocyte studies with in vivo or ex vivo models could enhance our understanding of DOX-induced cardiotoxicity and its clinical implications. Further investigation is needed to unravel the specific mechanisms these molecular changes contribute to DOX-induced cardiac dysfunction and explore their potential as diagnostic biomarkers and therapeutic targets.

## 4. Materials and Methods

### 4.1. Isolation of Ventricular Cardiomyocytes from Guinea Pig (Cavia porcellus)

Ventricular cardiomyocytes were isolated from adult female Hartley guinea pigs (*Cavia porcellus*) weighing between 280 and 320 g. The isolation process utilized the Langendorff-type retrograde perfusion system combined with enzymatic dissociation employing collagenase, pronase, and proteinase K, as outlined by Gómez et al. [24]. Before thoracotomy and cardiotomy, we anesthetized the guinea pigs with pentobarbital (50 mg/kg intraperitoneally). Following excision, the hearts were promptly transferred to a Langendorff-type retrograde perfusion system containing TC-199 culture medium and heparin (1000 U/mL) for 2–3 min to eliminate blood and initiate rhythmic contractility. Subsequently, cardiomyocytes were isolated, and cell count was determined using the trypan blue exclusion assay. The isolated cardiomyocytes were then exposed to 10 μM DOX and incubated at room temperature for 22 h.

The Institutional Committee for the Care and Use of Animals (CICUAL) approved the protocol for obtaining primary cultures of cardiomyocytes from Guinea pigs. We sourced the animals from the hearts of Hartley guinea pigs (*Cavia porcellus*) bred in specialized research animal facilities that adhere to rigorous animal welfare standards approved by CICUAL. CICUAL evaluated and approved the ethical and technical relevance of animal protocol on animal protection and experimentation to safeguard animal health, welfare, and humane treatment during the evaluation process conducted at the National Institute of Health (INS) of Colombia. The specific code number and date of approval issued by R-06-November-2023-CICUAL.

### 4.2. Evaluation of the Length and Percentage of Cell Shortening of Cardiomyocytes

The length and percentage of shortening of cardiomyocytes were assessed utilizing images captured by light microscopy using the Cytation™ equipment. These images were subsequently analyzed employing the free software ImageJ Fiji 1.54f. Measurement of the length of cardiomyocytes was conducted, and the percentage of cell shortening was calculated. The percentage of cardiomyocyte shortening was determined as the change in length relative to the initial length.

### 4.3. Evaluation of ROS Levels, Intracellular Calcium, and Mitochondrial Membrane Potential

We evaluated R.O.S production using the reagent Dihydroethidium (DHE). We evaluated mitochondrial membrane potential using the JC-1 reagent from Invitrogen and measured intracellular calcium levels using the FLUO 4 AM reagent. We evaluated it under a fluorescence microscope and obtained photographic records. Subsequently, we analyzed the photographs using the free ImageJ Fiji software. We quantified mean fluorescence intensities for DHE, FLUO 4 AM, and JC1 and performed statistical analysis using the two-way ANOVA parametric test (*p* < 0.05, *n* = 10 cardiomyocytes per cluster).

### 4.4. RNA Extraction, Small RNA Sequencing, and Analysis

Total RNA extraction was conducted on isolated guinea pig ventricular cardiomyocytes exposed independently to 10 μM of DOX and to the vehicle control (DMSO) for 22 h at room temperature. We used Trizol reagent for extraction following the manufacturer’s guidelines (Invitrogen). The extracted RNA was resuspended in H2O DEPC (diethyl pyrocarbonate, Sigma–Aldrich) and quantified via spectrophotometry using the Nanodrop 2000 (Thermo Fisher Scientific, Norristown, PA, USA).

Subsequently, we performed small RNA sequencing using Illumina technology (Illumina, San Diego, CA, USA) to sequence small RNAs. We estimated the RNA integrity number (RIN) parameter using the Agilent RNA Screen Tape System (Illumina). A small RNA library was constructed using the TruSeq Small R.N.A. Library Prep Kit (Illumina Inc., San Diego, CA, USA). We performed quality control and normalized the readings obtained using the BaseSpace^®^ App. We aligned the reads against the reference genome for *Homo sapiens*. We registered the data at the Gene Expression Omnibus-GEO-NCBI with the access number GSE263950 (https://www.ncbi.nlm.nih.gov/geo/query/acc.cgi?acc=GSE263950) (accessed on 15 April 2024).

### 4.5. RT-qPCR-Stem-Loop for miRNAs and RT-PCR for mRNAs

We selected five miRNAs to validate their expression using RT-qPCR with a stem-loop strategy. We used the SuperScript IV First-Strand Synthesis System (Thermo Fisher Scientific, Waltham, MA, USA) with 45 ng of enriched RNA for the RT assays. The reactions were performed in a BIO-RAD C1000 thermocycler under the following conditions: 30 min at 16 °C, followed by 60 cycles of 30 s at 30 °C, 30 s at 42 °C, and 1 min at 50 °C, concluding with a 5 min incubation at 85 °C. We used 1400 ng of cDNA and 250 nM of primers for the qPCR of miRNAs, employing the DyNAmo HS SYBR Green Kit (Thermo Fisher Scientific). The qPCR was carried out in a BIO-RAD CFX96 thermocycler with a program set to 3 min at 95 °C, followed by 40 cycles of 15 s at 95 °C for denaturation, 50 s at 61 °C for annealing, and 1 min at 72 °C for extension, with a final 7 min extension at 72 °C.

We synthesized cDNA from 500 ng of total RNA using the SuperScript IV First-Strand Synthesis System (Thermo Fisher Scientific) for mRNA RT-qPCR. The reaction profile in the BIO-RAD thermal cycler was 5 min at 65 °C, 10 min at 50 °C, and 15 min at 70 °C. For qPCR, we used 1400 ng of cDNA and 250 nM of sense and antisense primers corresponding to each evaluated mRNA, with the GoTaq^®^ Green Master Mix (Promega). Amplification occurred in a BIO-RAD CFX96 thermal cycler with an initial 2 min at 95 °C to activate the polymerase, followed by 35 cycles of 10 s at 96 °C for denaturation, 20 s at the primer-specific annealing temperature, and 30 s at 72 °C for extension. Melting curve analysis was performed from 55 to 95 °C using the CFX Maestro 2.3 Software (BIO-RAD) for both miRNA and mRNA qPCR assays.

To analyze differential expression quantitatively, we utilized the Relative Expression Ratio (rER) model to compare the expression levels of miRNAs and mRNAs, adjusting for PCR efficiency [72]. We used succinate dehydrogenase (SDH) as the reference gene for mRNA and U6 snRNA for miRNA.

### 4.6. Prediction of Molecular Targets (mRNAs) of microRNAs by CSmiRTar

We employed CSmiRTar (http://cosbi4.ee.ncku.edu.tw/CSmiRTar/search) (accessed on 27 March 2024) to predict potential molecular targets (mRNAs) of microRNAs [73]. Specifically, we focused on microRNAs with differential expression identified through DESeq2 analysis. The predicted target genes were selected based on their support from at least two databases and an Average Normalized Score (ANS) of ≥ 0.2. This criterion ensured the reliability of the predicted targets. By utilizing this approach, we aimed to identify microRNAs that might be involved in modulating the expression of genes associated with the observed phenotypic changes.

### 4.7. Protein Expression by Western Blot

We isolated ventricular cardiomyocytes by first washing them with sterile Tyrode’s Solution. Then, we mechanically lysed them in the presence of RIPA buffer (Sigma-Aldrich) containing 1X Pierce Protease and Phosphatase Inhibitor (A32959) from Thermo Fischer Scientific. The lysed samples underwent centrifugation at 8000× *g* for 10 min at 4 °C. The supernatant containing the proteins was carefully collected. Protein concentration was quantified using a calibration curve constructed with bovine serum albumin and bicinchoninic acid (Pierce TM BCA Protein Assay Kit, Thermo Fisher Scientific) following the manufacturer’s recommendations. We loaded 30 µg of protein per sample onto a 10% SDS-PAGE gel and electrophoresed them under denaturing conditions. Proteins separated on the gel were electro-transferred onto a PVDF membrane (Millipore-Merck) using the Novex^®^ semi-dry system (Thermo Fisher Scientific) following the manufacturer’s recommendations. We blocked nonspecific binding sites on the PVDF membrane with a 1% (*w*/*v*) solution of polyvinylpyrrolidone (PVP-40) (Sigma-Aldrich, Burlington, MA, USA) in PBS-Tween 20 (Sigma-Aldrich). We incubated the membrane with primary antibodies against Kir 6.1 (S366-60), Kir 6.2 (PA5-99440), from Invitrogen SUR2A (ab174629) (Abcam), and β-tubulin (ZRB1124) (SIGMA). Then, we incubated the membrane with secondary antibodies labeled with horseradish peroxidase (HRP) anti-mouse (61-6520) and anti-rabbit (G-21234) from Invitrogen. Concurrently, we detected the cytoplasmic protein β-tubulin as a loading control. We completed immunodetection by chemiluminescence using the ECL Western blotting system (Amersham, Boston, MA, USA). We performed a densitometric analysis of the detected protein bands using Image Lab Software for P.C. Version 6.1.

### 4.8. miRNA Gene Regulatory Networks and Functional Enrichment Analysis

We performed a comprehensive functional analysis of differentially expressed miRNAs and the reported target gene using the ClueGO/CluePedia plugin from Cytoscape (ClueGO v2.5.10, CluePedia v. 1.5.10, Cytoscape v. 3.9.1 [35,36]. Briefly, we enriched the selected miRNAs with their validated target genes (CluePedia enrichment: All MTI for each miRNA, up to 300 genes, from databases mirTarBase (15.06.2016) and miRecords (2010.11.25), with a Score ≥ 0.6). We included proteins of interest in the list of miRNA–target interactions (MTIs) and experimentally evaluated them. We performed pathway enrichment (ClueGO enrichment: all evidence from KEGG (25.05.2022: 8135 genes), WikiPathways (23.02.2022: 7783 genes), and REACTOME Pathways (25.05.2022: 10,882 genes), with the following enrichment criteria: min. three genes or 4% min, enrichment/Depletion or two-sided hypergeometric test, with *p*-value significance < 0.05 corrected with Bonferroni step-down method, mid-*p*-values (all unique genes in selected ontologies as reference set), 60% for a cluster to be specific. We selected pathways and proteins of interest, developed a new CluePedia analysis, and analyzed the obtained MTI PPI network [74].

## 5. Conclusions

In conclusion, our study finds that DOX exposure changes specific miRNAs and mRNA expression of KATP subunits, FOXO1, GSK3β, and SIRT1, potentially implicating them in DOX-induced cardiotoxicity and related cardiac changes. Additionally, our results indicate the activation of signaling pathways in response to cardiomyocyte stress, and computational analyses reveal intricate regulation of miRNAs associated with cardiotoxic progression. These findings, supported by miRNA gene regulatory networks and functional enrichment analysis, suggest that DOX-induced cardiotoxicity disrupts biological processes linked to endogenous cardioprotective mechanisms. Further research must clarify their specific molecular changes in DOX-induced cardiac dysfunction and investigate their biomarkers diagnosis and therapeutic potentials.

## Figures and Tables

**Table 1:** List of miRNAs expression in Cardiomyocytes exposed to 10 µM DOX for 1 h. microRNA I.D. refers to the identification number of miRNA registers in miRBase; log2 fold change represents times of change in the expression; *p*-value corresponds to the statistical significance.

Cardiomyocytes Exposed to 10 µM DOX for 1 h Versus Unexposed Cardiomyocytes				
miRNA ID	Expression Level	Sequence	log2 (Fold Change)	*p*-Value
miR-27a-5p	Downregulated	AGCTTAGCTGATTGGTGAACT	−1.09	9.53 × 10^−3^
miR-181a-5p		CATTCAACGCTGTCGG	−1.05	1.44 × 10^−2^
miR-148b-5p		AGTTCTGTTATACACTCAG	−0.83	5.07 × 10^−2^
miR-181b-3p	Upreguladed	CACTGATCAATGAATGCAA	0.83	5.17 × 10^−2^
miR-127-5p		AGCTCAGAGGGCTCTGAT	0.86	3.55 × 10^−2^
miR-23b-3p		CACATTGCCAGGGA	1.00	1.54 × 10^−2^
let-7d-5p		AGGTAGTAGGTTGTATAGTTA	1.18	4.11 × 10^−2^
miR-99b-5p		CCCGTAGAACCGATCTTGTG	1.91	2.20 × 10^−8^

**Table 2:** List of miRNA expression in Cardiomyocytes exposed to 10 µM DOX for 22 h. MicroRNA ID refers to the identification number of the miRNA register in miRBase; log2 fold change represents the times of change in the expression; *p*-value corresponds to the statistical significance.

Cardiomyocytes Exposed to 10 µM DOX for 22 h Versus Unexposed Cardiomyocytes				
miRNA ID	Expression Level	Sequence	log2 (Fold Change)	*p*-Value
miR-99b-5p	Downregulated	CCCGTAGAACCGATCTTGTG	−1.46	4.49 × 10^−4^
miR-24-1-5p		TGCCTACTGAGCTGATATC	−0.82	2.59 × 10^−2^
miR-133a-1-3p		GGTCCCCTTCAATCAGCTGTT	−0.82	5.47 × 10^−2^
miR-378c-5p		ACTGGACTTGGAGTCAGAA	−0.81	5.98 × 10^−2^
miR-425-5p	Upregulated	TGACACGATCACTCCCGTTGT	0.89	3.90 × 10^−2^
miR-136-5p		TCCATTTGTTTTGATGATGG	1.00	2.01 × 10^−2^

## Data Availability

In this study, we analyzed available datasets. These data are accessible here (https://www.ncbi.nlm.nih.gov/geo/query/acc.cgi?acc=GSE263950 accessed on 15 April 2024).

## Supplementary Materials

The following supporting information can be downloaded at: https://www.mdpi.com/article/10.3390/ijms25105272/s1.
