# Supplementary material for: miRNA Expression Profiles in Isolated Ventricular Cardiomyocytes: Insights into Doxorubicin-Induced Cardiotoxicity
Source: Int J Mol Sci. 2024 May 12;25(10):5272. doi: 10.3390/ijms25105272 (PMC11121573; doi:10.3390/ijms25105272)
Supplement: Supplementary file 1 [file ijms-25-05272-s001.zip › Figures suplementarias.pdf]

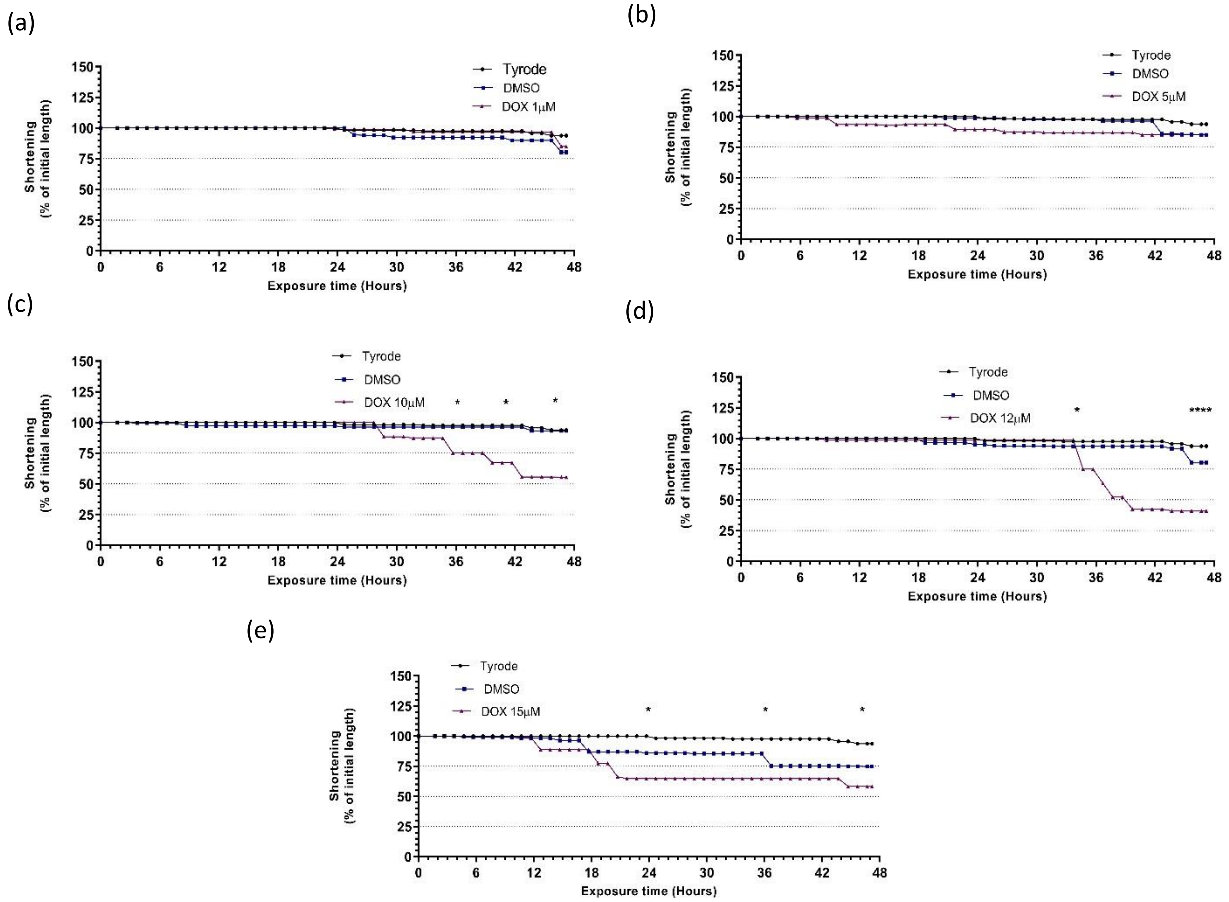

Figure S1. DOX induces the shortening of *Cavia porcellus* cardiomyocytes. We exposed isolated cardiomyocytes to DOX concentrations (1-15 μM). Shortening percentage of cardiomyocytes during 48 hours of exposure to a) 1 μM of DOX, b) 5 μM of DOX, c) 10 μM of DOX, d) 12 μM of DOX, e) 15 μM of DOX. Data were analyzed using a two-way ANOVA parametric statistical test ($p<0.05$, $n=10$ cardiomyocytes per group)—representative graphs of three experiments.

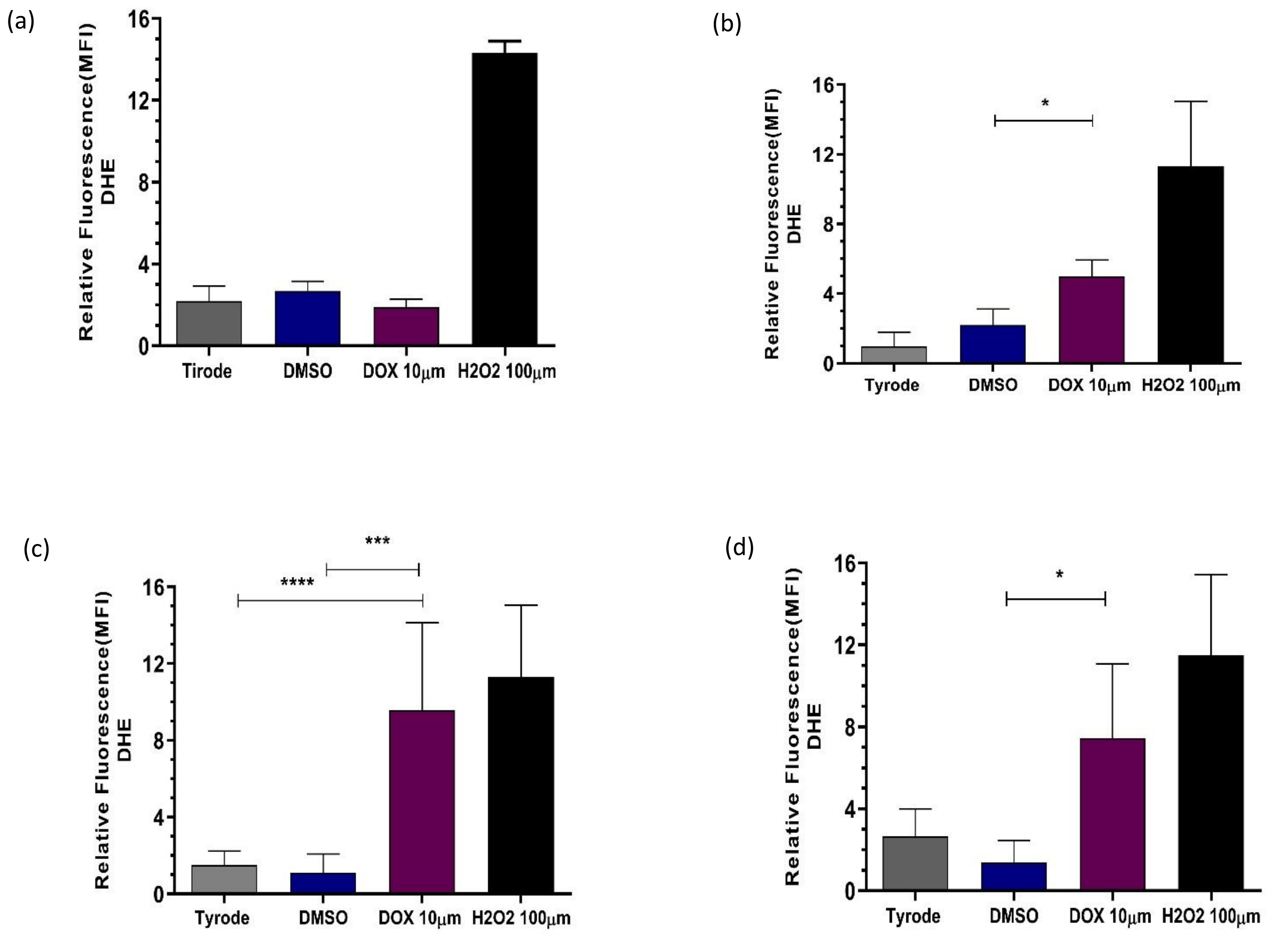

Figure S2. DOX induces an increase in ROS level in *Cavia porcellus* cardiomyocytes. We exposed isolated cardiomyocytes to DOX 10 μM for varying durations (1-30 hours). ROS production in cardiomyocytes exposed to DOX at: a) 1 hour, b) 12 hours c) 24 hours d) 30 hours. Data were analyzed using two-way ANOVA parametric statistical test ($p<0.05$, $n=10$ cardiomyocytes per group)—representative graphs of three different experiments.

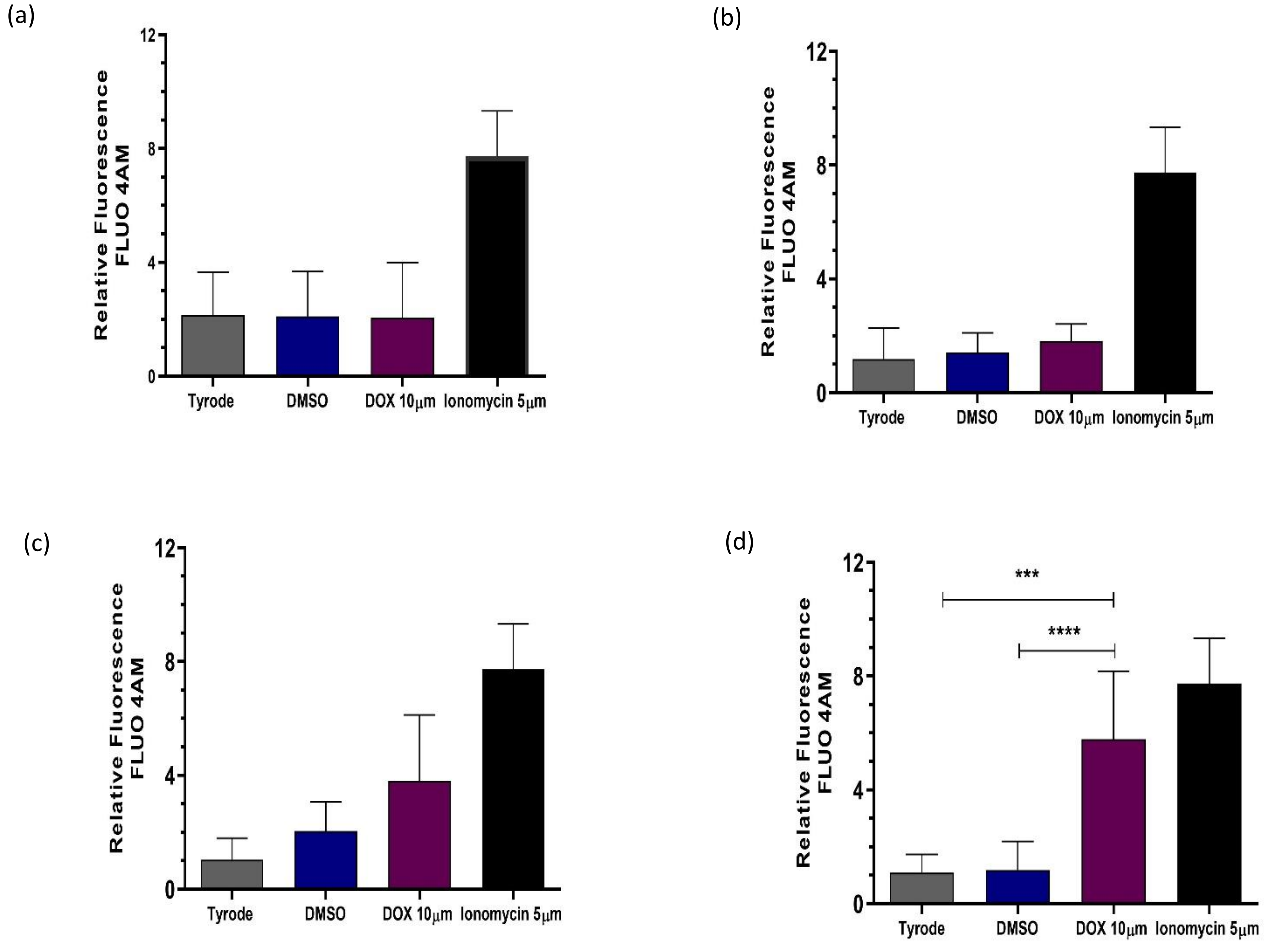

Figure S3. Exposure to DOX increases intracellular (Ca2+) levels in Cavia porcellus cardiomyocytes. We exposed isolated cardiomyocytes to DOX 10 μM, and we evaluated intracellular calcium levels for varying durations (1-30 hours). ROS levels analyzed with DHE in cardiomyocytes exposed to DOX at: a) 1 hour, b) 12 hours c) 24 hours d) 30 hours. Data were analyzed using two-way ANOVA parametric statistical test ($p<0.05$, $n=10$ cardiomyocytes per group)—representative graphs of three different experiments.

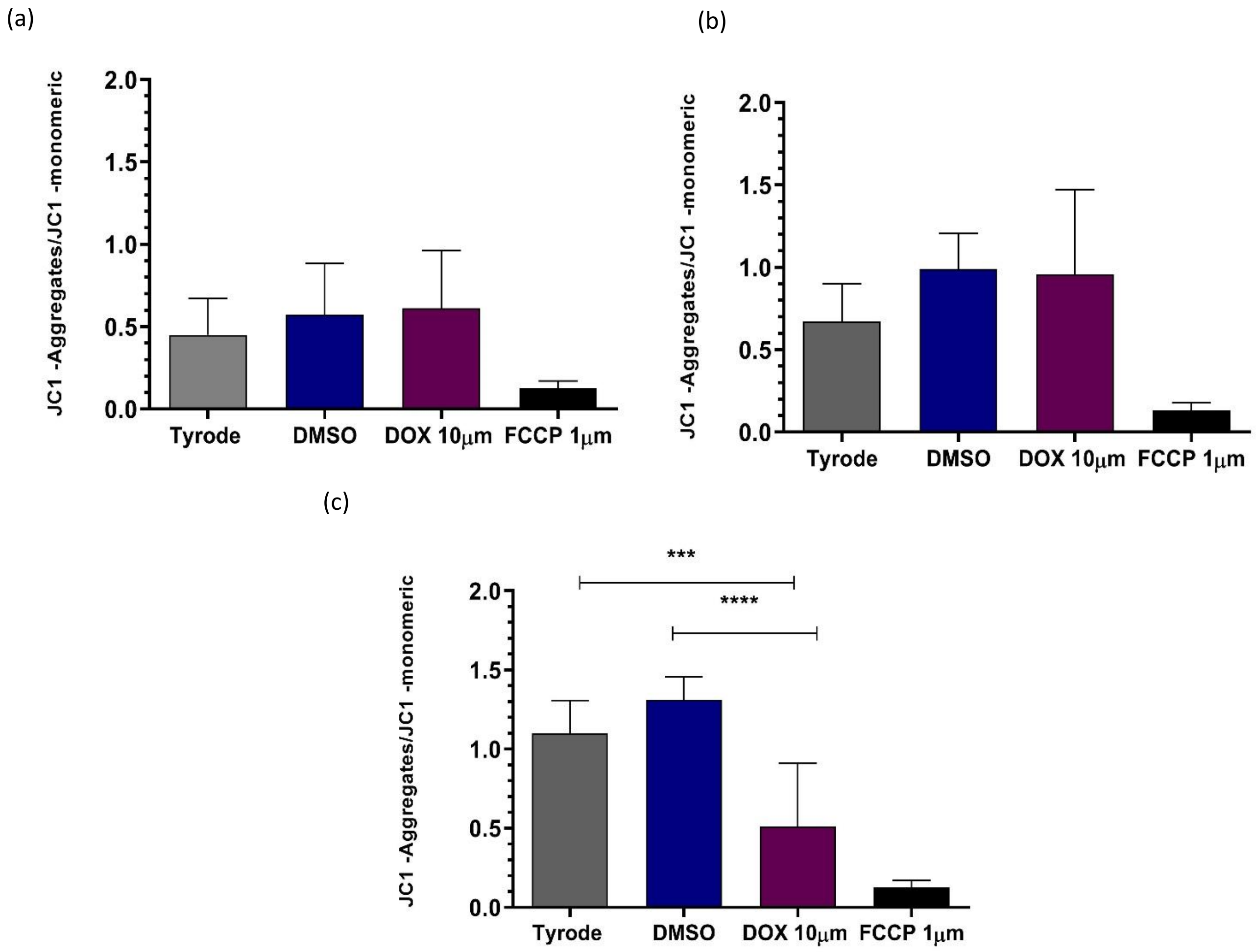

Figure S4. Alteration of mitochondrial membrane potential by exposure to $10\mu M$ DOXO. We exposed isolated cardiomyocytes to DOX at the indicated concentration, and we evaluated the potential membrane for varying durations (1-30 hours). Mitochondrial membrane potential ($\Delta\Psi m$) analyzed with JC1 in cardiomyocytes exposed to DOX at: a) 1 hour, b) 12 hours c) 30 hours. Data were analyzed using two-way ANOVA parametric statistical test ($p<0.05$, $n=10$ cardiomyocytes per group)—representative graphs of three different experiments.
